# *Babesia microti* in Rodents from Different Habitats of Lithuania

**DOI:** 10.3390/ani11061707

**Published:** 2021-06-07

**Authors:** Dalytė Mardosaitė-Busaitienė, Jana Radzijevskaja, Linas Balčiauskas, Algimantas Paulauskas

**Affiliations:** 1 Faculty of Natural Sciences, Vytautas Magnus University, LT-44243 Kaunas, Lithuania; dalyte.mardosaite-busaitiene@vdu.lt (D.M.-B.); algimantas.paulauskas@vdu.lt (A.P.); 2 Laboratory of Mammalian Ecology, Nature Research Centre, LT-08412 Vilnius, Lithuania; linas.balciauskas@gamtc.lt

**Keywords:** 18S rRNA, *Babesia*, rodents, voles, mice, Lithuania

## Abstract

**Simple Summary:**

*Babesia microti*, the causative agent of human babesiosis, is an intraerythrocytic protozoan parasite, that circulates among small rodents and ixodid ticks in many countries worldwide. Zoonotic and non-zoonotic *B. microti* strains have been identified in rodent populations in Europe. Analyzing eight species of small rodents collected from different habitats (meadows, forests and their ecotones) in Lithuania, we checked for the presence of *B. microti* and found the highest infection prevalence to be in *Microtus oeconomus* and *Microtus agrestis* rodents. Of note, this study also detected the first reported cases of *Babesia* parasites in *Micromys minutus* mice. In term of habitat, the highest prevalence of *Babesia* parasites was detected in rodents trapped in meadows. Our results demonstrate that rodents, especially *Microtus* voles, can play an important role in the circulation of the zoonotic *B. microti* ‘Jena/Germany’ strain in Lithuania.

**Abstract:**

*Babesia microti* (Aconoidasida: Piroplasmida) (Franca, 1910) is an emerging tick-borne parasite with rodents serving as the considered reservoir host. However, the distribution of *B. microti* in Europe is insufficiently characterized. Based on the sample of 1180 rodents from 19 study sites in Lithuania, the objectives of this study were: (1) to investigate the presence of *Babesia* parasites in eight species of rodents, (2) to determine the prevalence of *Babesia* parasites in rodents from different habitats, and (3) to characterize the detected *Babesia* strains using partial sequencing of the 18S rRNR gene. *Babesia* DNA was detected in 2.8% rodents. The highest prevalence of *Babesia* was found in *Microtus oeconomus* (14.5%) and *Microtus agrestis* (7.1%) followed by *Clethrionomys glareolus* (2.3%), *Apodemus flavicollis* (2.2%) and *Micromys minutus* (1.3%). In *M.* *minutus*, *Babesia* was identified for the first time. The prevalence of *Babesia*-infected rodents was higher in the meadow (5.67%) than in the ecotone (1.69%) and forest (0.31%) habitats. The sequence analysis of the partial 18S rRNA gene reveals that *Babesia* isolates derived from rodents were 99–100% identical to human pathogenic *B. microti* ‘Jena/Germany’ strain.

## 1. Introduction

Babesiae are emerging tick-borne protozoan parasites circulating in many countries worldwide in vertebrate hosts and vectors. The *Babesia* species including *Babesia microti*, *Babesia divergens*, *B. divergens*-like, *Babesia venatorum* and *Babesia duncani* are known to cause infection in humans. In Europe, Asia and North America respectively, the main vectors of zoonotic *Babesia* species are *Ixodes ricinus*, *Ixodes persulcatus* and *Ixodes scapularis* ticks. [1]. *B. microti* is the main causative agent of human babesiosis, especially in North America [2]. In Europe however, human babesiosis cases are less frequently reported and mostly related to *B. divergens*, *B. divergens*-like and *B. venatorum* [3]. However, a few cases of human babesiosis resulting from *B. microti* have also been reported in Europe [4,5,6]. To the best of our knowledge, no cases of human babesiosis have been documented in Lithuania.

The common vole (*Microtis arvalis*), field vole (*Microtus agrestis*) and root vole (*Microtus oeconomus*) are microtine rodents that play important roles in the circulation of *B. microti* in Europe [7,8]. *B. microti* infection was also detected in yellow-necked mouse (*Apodemus flavicollis*), striped field mouse (*Apodemus agrarius*), wood mouse (*Apodemus sylvaticus*) and bank vole (*Clethrionomys glareolus*), which are the main hosts for the immature stages of *Ixodes* ticks [7,9,10,11]. In general, *Ixodes trianguliceps* (with all three developmental stages feeding on rodents and does not bite humans) is the main vector of *B. microti* [12,13], while *I. ricinus* (with larvae and nymphs feeding on rodents) would only serve as a bridge vector of *B. microti* [9,14,15].

Molecular phylogenetic analysis demonstrated that *B. microti* consisting of genetically diverse isolates that belong to different clades [16]. *B. microti* isolates from rodents are subdivided within these clades into the non-zoonotic and zoonotic strains [17,18]. Different strains of *B. microti* have been reported in rodents in Slovenia, Croatia, Poland, Finland, Germany, Slovakia and France [7,9,12,19,20,21,22]. Various *B. microti* strains may circulate in rodent community at the same time [7]. However, distributions of *B. microti* strains in Europe are still insufficiently characterized.

The aims of the present study were: (1) to investigate the presence of *Babesia* parasites in eight species of Lithuanian rodents, (2) to determine the prevalence of *Babesia* parasites in rodents from meadows, forests and their ecotones, and (3) to characterize the detected *B. microti* strains using partial sequencing of 18S rRNR gene.

## 2. Materials and Methods

### 2.1. Study Sites

**Figure 1:**
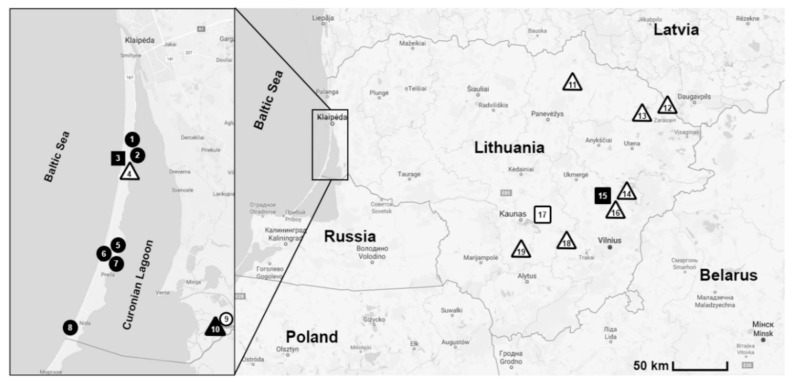
Rodent trapping sites in Lithuania, 2013–2017. ∆/▲—forests; ○/●—meadows; □/■—ecotones; ∆/○/□—rodents negative for *Babesia* parasites; ▲/●/■—rodents infected with *Babesia* parasites. Map was created using Open street map data (open source https://www.openstreetmap.org, accessed on 22 April 2020).

Rodents were trapped in 19 locations of different habitats in western (Curonian Spit; sites 1–8 and Nemunas River Delta; sites 9–10) and eastern (sites 11–19) parts of Lithuania during 2013–2017 (Figure 1). Rodents were captured in meadows, forests and their ecotones. Rodent sampling in the Curonian Spit was conducted in the coastal meadows (sites 1, 2, 5–8), mixed forests (site 4) and meadow-mixed forest ecotone (site 3). In the Nemunas River Delta, the trapping was conducted in two habitats: in a flooded meadow (site 9) and in a spring-flooded black alder stands forest (site 10).

Eastern Lithuania was represented by different habitats—mixed forests (sites 11,13, 14, 16,19), mixed forest-meadow ecotones (sites 15, 17), and deciduous forests, one of them in peninsula of Lukštas lake (site 12) and the other on an island in an artificial water body, Elektrėnai Reservoir (site 18) (Table 1).

### 2.2. Rodent Trapping

Rodents were trapped by using live or snap traps baited with bread immersed in unrefined sunflower oil. One trapping session consist of three days. The traps were checked two times per day [23]. All rodents were identified to species level and gender morphologically and under dissection, with specimens of *Microtus* voles identified by their teeth [24].

### 2.3. Molecular Analyses

DNA from rodent spleen was extracted using Genomic DNA Purification Kit (Thermo Fisher Scientific, Vilnius, Lithuania), according to the manufacturer’s protocol. The presence of *Babesia* pathogens were conducted through the amplification of the 330 bp fragment of the 18S rRNA gene in nested PCR using two primer sets BS1/BS2 and PiroA/PiroC as described by Rar et al. [25,26]. The primary PCR reaction was carried out in a 20 μL final volume containing: 1 × PCR buffer, 2 mM MgCl_2_, 0.2 mM dNTPs, 10 pmol of each primer, 2 U Taq DNA polymerase (Thermo Fisher Scientific, Lithuania), double-distilled water and 2 μL of DNA template. Reaction was performed according to the conditions: initial denaturation at 94 °C for 3 min, 35 cycles: denaturation at 94 °C for 60 s, annealing at 58 °C for 60 s and extension at 72 °C for 90 s, and final extension step at 72 °C for 3 min. In the second PCR, the reaction mix was similarly prepared as it was in the first step, with exception that instead of the DNA, 1 μL of the PCR product was added. The PCR conditions were: initial denaturation at 94 °C for 3 min, followed by 35 cycles: denaturation at 94 °C for 60 s, annealing at 64 °C for 60 s, and extension at 72 °C for 90 s. The final extending was at 72 °C for 3 min. In each PCR run negative (double-distilled water) and positive (DNA of *Babesia* positive ticks, infection confirmed by sequencing) controls were used. The PCR products were analyzed by horizontal electrophoresis in 1.5% agarose gel and visualized with ethidium bromide solution (20 ng/μL) using ultra-violet transilluminator UVP GelDoc-It 310 model (Ultra-Violet Products Ltd., Cambridge, UK). The good quality PCR products of *Babesia*-positive samples were extracted from agarose gel. GenJet PCR purification kit (Thermo Fisher Scientific, Lithuania) was used for purification and, after preparation, samples were sent for direct sequencing by Sanger method to Macrogen Europe company (Amsterdam, The Netherlands).

The partial 18S rRNA sequences were analyzed using MEGA X software package, version 10.0.5. [27] and compared with the sequence data available in NCBI GenBank database using the NCBI BLAST^®^ software (http://blast.ncbi.nlm.nih.gov, accessed on 6 June 2020). A phylogenetic tree was constructed by applying maximum-likelihood (ML) method implemented with Tamura-Nei model. Partial 18S rRNA sequences for representative samples were submitted to GenBank under the accession numbers: MT745579 to MT745583.

### 2.4. Statistical Analysis

The between-species and between-location differences in the prevalence of *Babesia* infection were tested. For these differences, we used Fisher’s exact test and the Mantel–Haenszel common odds ratio estimate. Calculations were performed in SPSS software version 22 (IBM SPSS, Chicago, IL, USA), using 95% confidence intervals. We assessed the prevalence of *Babesia* in all investigated rodent species; calculations were performed in OpenEpi software [28]. 95% CI for prevalence was calculated according to the Wilson method [29]. We tested the significance of differences in the prevalence between species and between habitats. These calculations were performed in WinPepi, ver. 11.39. We used the chi-squared test with Upton’s approximation for small and medium sample sizes. To express the effect size, we used adjusted Cohen’s w [30]. In all tests, *p* < 0.05 was considered significant.

## 3. Results

We analyzed 1180 rodent individuals, belonging to eight species, best represented by *A. flavicollis* and *C. glareolus* (Table 1). *Babesia* infected rodents were trapped in nine out of 19 sampling locations (Figure 1). *A. flavicollis* was the dominant trapped rodent species in Curonian Spit (70.9%; 409/577) with the prevalence of infection ranging in four locations (where the infected rodents were captured) from 1.1% to 8.1%. *Babesia* infected *C. glareolus* were found in six of the fifteen sampling locations: with the overall prevalence of infection estimated at 6.5% on the Curonian Spit, 3% in the Nemunas River Delta and 1.3% in the eastern part of the country. *Babesia* infected *M. oeconomus* and *M. agrestis* were found in three and one sampling locations in the Curonian Spit, respectively. One *Babesia* infected harvest mouse (*Micromys minutus*) specimen was found in one location in the Curonian Spit (site 1) (Table 1). All trapped house mice (*Mus musculus*), *A. agrarius* and *M. arvalis* were not infected.

### 3.1. Prevalence of Babesia Parasites in Various Rodent Species

**Figure 2:**
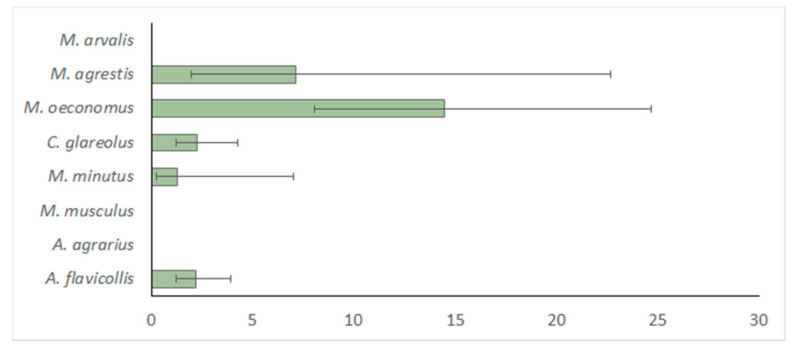
Prevalence (in %, bars represent 95% CI) of *Babesia* in the eight rodent species, irrespective to the habitat.

A total of 33 (2.8%, CI = 1.74–4.23%) out of 1180 DNA samples of rodents were positive for *Babesia* DNA. The species-based differences of prevalence of *Babesia* parasites were significant (14.5%; OR, 3.6; 95% CI, 1.330–9.625; *p* < 0.012) and are presented in Figure 2. The highest prevalence of *Babesia* was found in *M. oeconomus* and *M. agrestis* (14.5% vs. 7.1%, χ2 = 0.98, NS; Cohen’s w = 0.101, small effect size). The prevalence in *M. oeconomus* was significantly higher than that in *C. glareolus* (χ2 = 23.1, w = 0.221), *A. flavicollis* (χ2 = 25.7; w = 0.213) and *M. minutus* (χ2 = 9.0; w = 0.250). All differences are significant at *p* < 0.001, effect sizes medium.

### 3.2. Habitat-Based Differences

In general, the highest prevalence of *Babesia* parasites was characteristic to rodents, trapped in meadows (5.67%, CI = 3.87–8.23%), exceeding that in forests (0.31%, CI = 0.05–1.72%), with intermediate prevalence values observed in ecotones (1.69%, CI = 0.82–3.46%). The differences between meadow-forest (χ2 = 16.4, *p* < 0.001) and meadow-ecotone (χ2 = 9.3, *p* = 0.002) were highly significant with medium effect size, while that of forest-ecotone (χ2 = 3.3, *p* = 0.07) had only a trend without effect (w = 0.067).

**Figure 3:**
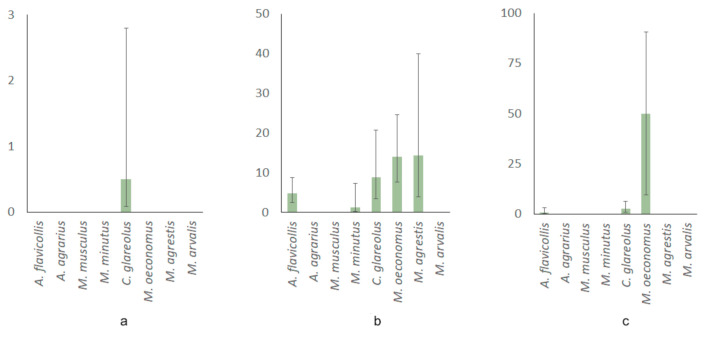
Habitat-based differences of prevalence (in %, bars represent 95% CI) of *Babesia* in the eight rodent species: (**a**) forests, (**b**) meadows, (**c**) ecotones.

In the forests, only *C. glareolus* was infected by *Babesia* with low prevalence (Figure 3a). In the meadows, minimum observed prevalence of *Babesia* in *M. minutus* (Figure 3b) was significantly exceeded by prevalence in *M. oeconomus* (χ2 = 8.2, p < 0.01; w = 0.245, medium effect size), *M. agrestis* (χ2 = 5.9, *p* = 0.015; w = 0.261, medium effect) and *C. glareolus* (χ2 = 3.9, *p* < 0.05; w = 0.262, small effect size). Other differences of *Babesia* prevalence between rodent species in meadows were not significant. In the forest-meadow ecotone, prevalence of *Babesia* in *M. oeconomus* was higher than in other species, despite minimum sample size (Figure 3c).

### 3.3. Molecular Characterization of Babesia Isolates

**Figure 4:**
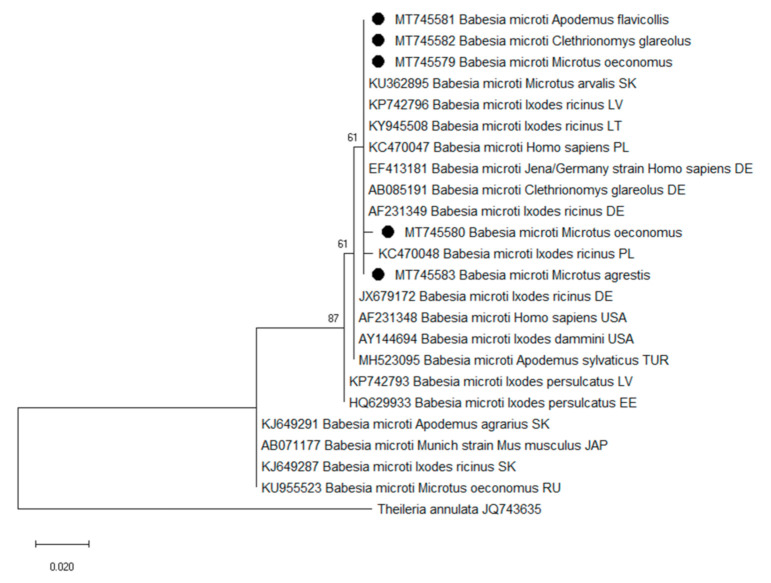
Phylogenetic tree of the partial 18S rRNA gene of *Babesia microti* inferred by ML method, the Tamura–Nei model and bootstrap analysis of 1000 replicates. Marked with dark circle are samples sequenced in the present study. Sequences MT745579 and MT745581 are representative of six and five other samples obtained in this study (from *A. flavicollis* and *M. oeconomus*), respectively. Sequences MT745582 and MT745583 are representative of two and one other samples sequenced in the present study (from *C. glareolus* and *M. agrestis*), respectively.

A total of 19 18S rRNA sequences derived from four rodent species *A. flavicollis* (n = 7), *C. glareolus* (n = 3), *M. oeconomus* (n = 7) and *M. agrestis* (n = 2) were analyzed. The sequence analysis of the partial 18S rRNA gene revealed that *Babesia* isolates derived from rodents were 99–100% identical to *B. microti* ‘Jena/Germany’ strain (GenBank: KC470047; EF413181). Two genotypes with one nucleotide difference were detected in *M. oeconomus* (Figure 4).

## 4. Discussion

In this study, *Babesia* DNA was detected in 33 of 1180 (2.8%) spleen tissue samples of five small rodent species. The overall prevalence of *Babesia* varied among rodent species with the highest prevalence detected in voles *M. oeconomus* (14.5%) and *M. agrestis* (7.1%) (Table 1). *Babesia* infected *M. oeconomus* and *M. agrestis* have been found in north-eastern Poland with the 39.5% (30/76) and 17.7% (3/17) prevalence of infection, respectively [8]. These figures are almost three times higher compered than that obtained in this study. A high prevalence of *Babesia* spp. in *M. agrestis* has been reported in Austria 30.4% (14/46) [31] and the United Kingdom 27.9% (671/2402) [14], while Šebek [32] reported a much lower 0.5% (1/218) prevalence of infection in this rodent species in the former Czechoslovakia. The low overall prevalence of *Babesia* in this study was detected in *C. glareolus* (2.3%). In other European countries, the prevalence of *Babesia* infection in *C. glareolus* varied: 39.7% (60/151) reported in Finland [21], 15.9% (60/151) in Slovenia [12], 11.9% (59/495) in north-eastern Poland [33], 6.1% (3/49) in Croatia [19,34], 6% (25/405) in the Netherlands [11], 0.8% (4/498) in Slovakia [7,35], 0.68% (1/147) in France [22], 0.03% (11/396) in Germany [9].

*Babesia* parasites were found with low prevalence in mice *A. flavicollis* (2.2%) and *M.* *minutus* (1.3%) irrespective of the study sites. In line with our results, a low prevalence of *Babesia* in *A. flavicollis* has been reported in Slovakia (1.7%; 12/706; [7,35]) and in Germany (0.01%; 1/178; [9]). However, higher *Babesia* infection rate detected in *A. flavicollis* was documented in Croatia 16.9% (11/65) [19,34], in north-eastern Poland 13.1% (8/61) [20] and in Slovenia 11.8% (15/127) [12]. To the best of our knowledge, our study is the first report of *Babesia* infection in *M. minutus*.

Although, *Babesia* parasites were not found in *A. agrarius* and *M. arvalis* in this study, it was detected in these rodent species trapped in other locations in Lithuania, with a prevalence of 2.1% and 9.1%, respectively [36].

In the present study, a significantly higher overall prevalence of *Babesia* among investigated areas was found on the Curonian Spit (4.9%, 28/577; OR, 3.3; 95% CI, 2.346–4.720; *p* < 0.000) with the highest prevalence of infection (among all locations examined) detected in coastal meadow, site 6 (14.3%, OR, 0.35; 95% CI, 0.173–0.708; *p* < 0.004). In line with this, five various rodent species have been found positive with *Babesia* in seven out of eight locations on the Curonian Spit (Table 1). The detected differences in the prevalence of *Babesia* parasites in the investigated locations might be explained by habitat factors: the highest infection prevalence was detected in the coastal meadows habitat (which on the Curonian Spit most frequent), and additionally by the fact that examined rodents trapped in the Curonian Spit were frequently infested with immature *I. ricinus* (mostly larvae) [37]. The overall prevalence of infestation with immature *I. ricinus* varied between rodent hosts and was highest for *A. flavicollis* (56%). The mean intensity of infestation with *I. ricinus* was 5.2 per rodent hosts (5.6 in *A. flavicollis,* 3.3 in *M. minutus*, 3.0 in *M. oeconomus*, 3.0 in *M. arvalis* and 2.0 in *C. glareolus*) (personal authors data).

Worldwide, seven *B. microti* strains—‘USA’, ‘Hobetsu’ (‘Otsu’), ‘Nagano’, ‘Kobe’, ‘Jena/Germany’, ‘Munich’ and ‘Baltic’ were identified [18,20,38,39,40]. In Europe, from those, four *B. microti* strains have been detected: the zoonotic ‘Jena/Germany’ and ‘USA’ strains, and non-zoonotic ‘Munich’ strain reported in *Ixodes* ticks and rodents [7,8,9,20,22,33] and the ‘Baltic’ strain detected in *I. persulcatus* collected from Estonia and Latvia [39,41] which pathogenicity for human is not known.

The zoonotic ‘Jena/Germany’ strain has been detected in *A. flavicollis*, *A. agrarius*, *M. arvalis* and *C. glareolus* from Slovakia [7,35], in *M. oeconomus*, *M. arvalis* and *M. agrestis* from Poland [8], in *A. flavicollis*, *C. glareolus* and *M. arvalis* from Germany [9] and in *A. flavicollis* and *C. glareolus* from central Croatia [19], while zoonotic *B. microti* ‘USA’ strain was detected in microtine rodents from north-eastern Poland [33]. In this study, the *B. microti* ‘Jena/Germany’ strains were detected in *A. flavicollis* mice and three voles species—*M. oeconomus*, *M. agrestis* and *C. glareolus*. In previous studies, the zoonotic ‘Jena/Germany’ strains were detected in *I. ricinus* ticks in Europe, including Baltic countries [39,41,42]. Autochthonous cases of human babesiosis due to the *B. microti* ‘Jena/Germany’ strain have been reported in Germany [4] and Poland [5]. Worldwide, most of the human babesiosis cases have been related to the zoonotic *B. microti* ‘USA’ strain. A lower virulence of European *B. microti* strains compared to those circulating in North America may be the reason of a lack of recognized human cases associated with European *B. microti* strains, despite human exposure to infectious tick bites in this continent. [43].

The non-zoonotic *B. microti* ‘Munich’ strain has been found in *C. glareolus* from Slovakia [7], in *A. flavicollis* and *C. glareolus* from central Croatia [19] and in *C. glareolus* from Finland [21] and France [22]. As a general rule, the *B. microti* ‘Munich’ strain was not found outside of the *I. trianguliceps* distribution area (from Great Britain to Baikal). It is thought that *I. trianguliceps* ticks play an important role for the maintenance of the non-zoonotic *B. microti* ‘Munich’ strain to mammalian hosts [17]. In Lithuania, *I. trianguliceps* ticks are present and previously were found on *A. flavicollis* and *C. glareolus* rodents [44]. However, in the present study, trapped rodents were infested only with immature *I. ricinus*.

## 5. Conclusions

Our findings suggest that rodents, especially *Microtus* voles, play an important role in the circulation of the zoonotic *B. microti* ‘Jena/Germany’ strain in Lithuania. The highest prevalence of *Babesia* parasites was detected in rodents trapped in coastal meadows. This study also detected *Babesia* infection in *M. minutus,* the first recorded infection in this species to the best of the authors’ knowledge.

## Figures and Tables

**Table 1:** Prevalence of *Babesia* parasites in Lithuanian rodents, 2013–2017 (presented as n/N, %) ^1^.

No	Habitat	*A. flavicollis*	*A. agrarius*	*M. musculus*	*M. minutus*	*C. glareolus*	*M. oeconomus*	*M. agrestis*	*M. arvalis*	Total
1	coastal meadow	4/59 (6.8)			1/40 (2.5)	1/9	1/4		0/1	7/113 (6.2)
2	coastal meadow							2/2		2/2
3	forest-meadow ecotone	2/192 (1.1)			0/2	0/36	1/2	0/1		3/233 (1.3)
4	mixed forest	0/29								0/29
5	coastal meadow	0/33			0/26	1/5	0/2			1/66 (1.5)
6	coastal meadow	2/54 (3.7)			0/1	1/3	8/18 (44.5)		0/1	11/77 (14.3)
7	coastal meadow	3/37 (8.1)			0/4	0/8			0/2	3/51 (5.9)
8	coastal meadow	0/5				1/1				1/6
9	flooded meadows		0/52		0/3	0/19	0/40	0/12		0/126
10	flooded forest	0/5	0/7		0/1	1/14 (2.5)	0/2	0/9		1/38 (2.6)
11	mixed forest						0/1		0/13	0/14
12	deciduous forest	0/12	0/6			0/52				0/70
13	mixed forest	0/3				0/7				0/10
14	mixed forest	0/3				0/11				0/14
15	forest-meadow ecotone	0/20				4/117 (3.5)				4/137 (2.9)
16	mixed forest	0/6				0/14				0/20
17	forest-meadow ecotone	0/17	0/10	0/12				0/4		0/43
18	deciduous forest	0/2	0/7			0/88				0/97
19	mixed forest	0/22				0/12				0/34
	Total	11/499 (2.2)	0/82	0/12	1/77 (1.3)	9/396 (2.3)	10/69 (14.5)	2/28 (7.1)	0/17	33/1180 (2.8)

^1^ n, number of individuals infected; N, number of individuals tested; No, site number.

## Data Availability

Partial 18S rRNA sequences for representative samples were submitted to GenBank under the accession numbers: MT745579 to MT745583.
